# Association between weight loss and outcomes in patients undergoing atrial fibrillation ablation: a systematic review and dose–response meta-analysis

**DOI:** 10.1186/s12986-023-00724-5

**Published:** 2023-01-31

**Authors:** Huilei Zhao, Xiaozhong Li, Peng Yu, Menglu Liu, Jianyong Ma, Jingfeng Wang, Wengen Zhu, Xiao Liu

**Affiliations:** 1 Department of Anesthesiology, The Third Hospital of Nanchang, Nanchang, 330006 Jiangxi China; 2 Department of Cardiology, The Second Affiliated Hospital of Nanchang University, Nanchang, 330006 Jiangxi China; 3 Department of Endocrinology, The Second Affiliated Hospital of Nanchang University, Nanchang, 330006 Jiangxi China; 4 Department of Cardiology, Seventh People’s Hospital of Zhengzhou, Zhengzhou, 334000 Henan China; 5 Department of Pharmacology and Systems Physiology, University of Cincinnati College of Medicine, 45267 Cincinnati, USA; 6 Department of Cardiology, Sun Yat-Sen Memorial Hospital of Sun Yat-Sen University, Guangzhou, 510080 Guangdong China; 7 Guangdong Province Key Laboratory of Arrhythmia and Electrophysiology, Guangzhou, 510120 China; 8 Department of Cardiology, The First Affiliated Hospital of Sun Yat-Sen University, Guangzhou, 510080 Guangdong China

**Keywords:** Atrial fibrillation, Recurrence, Weight loss, Ablation

## Abstract

**Background:**

Obesity is an strong risk factor for atrial fibrillation (AF), and obesity can affect the prognosis of AF. However, the role of weight loss on outcomes after ablation remains unclear.

**Objectives:**

This study aims to determine the relationship between weight loss and outcomes in patients with AF ablation, as well as the potential dose–response relationship.

**Methods:**

The Cochrane Library, PubMed, and Embase databases were searched to identify studies that reported a relationship between weight loss and ablation up to August 17, 2021. Relative risks (RRs) were pooled using random-effects models.

**Results:**

One randomized, open-labeled clinical trial and seven cohort studies involving 1283 patients were included. The mean body mass index of all included studies was over 30 kg/m^2^. The clinical trial showed a non-significant benefit of weight loss intervention on AF recurrence (Odd risk [OR] = 1.02, 95% confidence interval [CI] 0.70–1.47). Meta-analysis based on observational studies showed that the recurrence rate of AF after ablation was significantly reduced (RR = 0.43, 95% CI 0.22–0.81, I^2^ = 97%) in relatively obese patients with weight loss compared with the control group. Each 10% reduction in weight was associated with a decreased risk of AF recurrence after ablation (RR = 0.54, 95% CI 0.33–0.88) with high statistical heterogeneity (I^2^ = 76%). An inverse linear association (P_non-linearity_ = 0.27) between AF relapse and increasing weight loss was found.

**Conclusions:**

Our results first suggest an inverse dose–response association between weight loss and risk of recurrent AF after ablation, with moderate certainty.

**Graphical Abstract:**

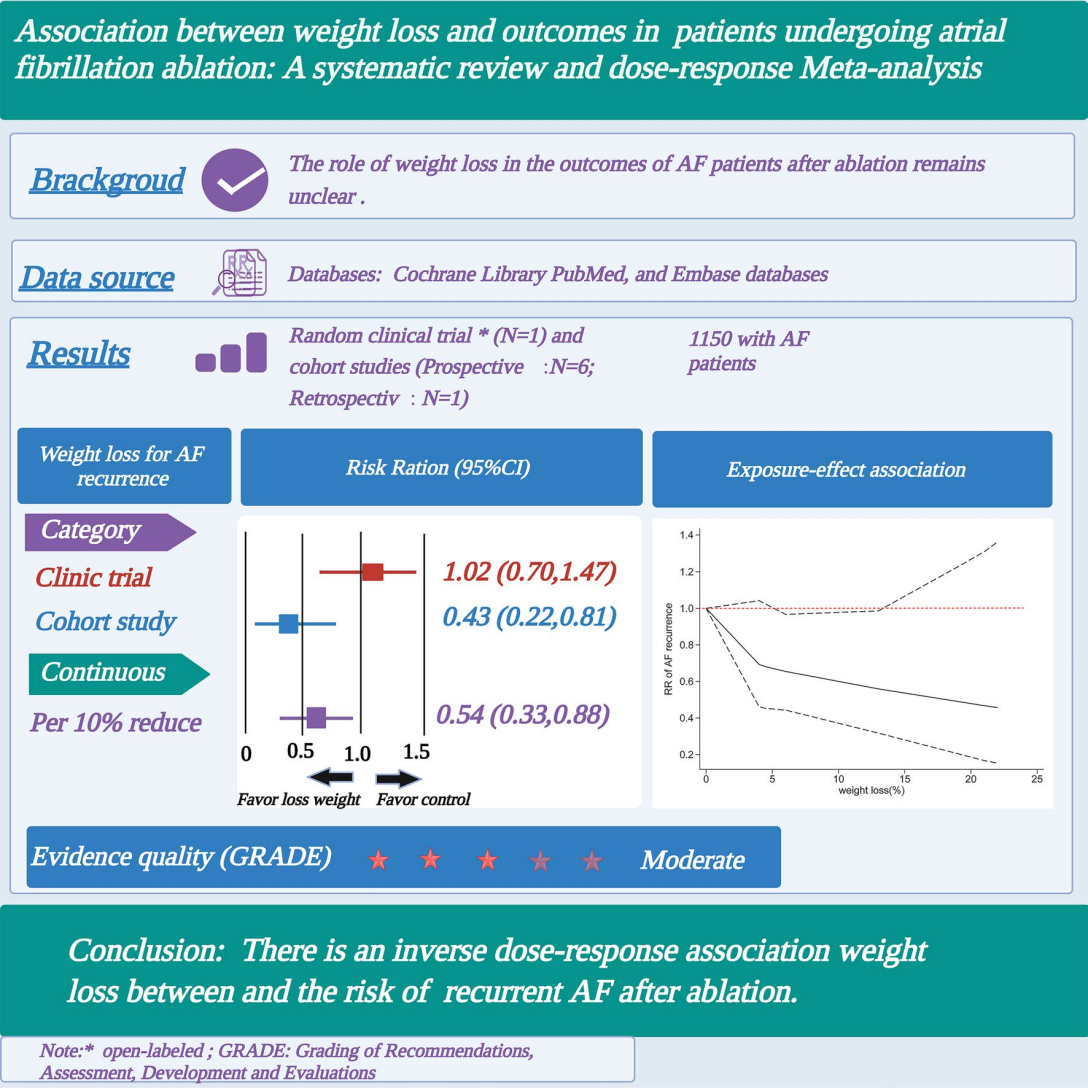

**Supplementary Information:**

The online version contains supplementary material available at 10.1186/s12986-023-00724-5.

## Introduction

Atrial fibrillation (AF) is the most common persistent arrhythmia in adults and contributes to an increased risk of stroke, dementia, and mortality. Several important risk factors have been identified in the occurrence and development of AF, such as hypertension, diabetes, obesity, and obstructive sleep apnea [1–5]. In the era that stresses everything from treatment to prevention, it is worth noting that effective management of AF requires not only the treatment of the disease itself but also the effective management of related comorbidities and risk factors.

Catheter ablation of AF is a recognized treatment method to prevent recurrence of AF, especially for drug-refractory symptomatic AF [6]. However, long-term reports of AF ablation showed that the success rate of ablation gradually declines over time [7–9]. Although there is an obesity paradox in AF patients, however, overweight and obesity have an adverse effect on cardiovascular prognosis in long-term follow-up [10, 11], and the guidelines [12] showed obesity increases the risk of AF progressively. But due to obstructive sleep apnea as a confounding factor, the guidelines did not demonstrate that obesity is a risk factor for AF recurrence ablation-post.

At present, research on controlling risk factors to reduce AF recurrence after ablation has become a hot topic. Obesity is a worldwide health problem and contributes to AF incidence after ablation [10, 11]. Some studies have shown that weight loss can reduce the AF incidence and burden of AF in obese populations [13, 14]. Similarly, some data suggest the benefit of weight loss in the prevention of AF recurrence after ablation; however, whether weight loss improves the outcomes after AF ablation remains unclear.

Thus, we conducted a meta-analysis to assess the relationship between weight loss and outcomes, including AF recurrence, AF symptom severity, AF burden, and quality of life.

## Method

This research was conducted in accordance with the guidelines of the 2020 Systematic Review and Meta-Analysis Preferred Reporting Project (PRISMA 2020) [15] (Additional file 1: Table S1).The protocol has been registered in PROSPERO (Prospective Registration for International System Evaluation.https://www.crd.york.ac.uk/prospero/display_record.php?RecordID=273682. CRD4202173680).

### Literature search

PubMed, Cochrane Library, Embase database and conference articles (American Heart Association: https://www.ahajournals.org/journal/circ, American College of Cardiology: https://www.jacc.org/ and European Society of Cardiology: https://www.escardio.org/) were searched by using the following MeSH to retrieve articles up to August 17, 2021, includes full text and conference abstracts, without language restrictions.

For patients: “atrial fibrillation”, “atrial flutter”, “atrial tachycardia”, and “ablation”.

For exposure/intervention: “weight loss”, “weight reduction”.

For outcomes: we did not apply any keywords for outcomes because all reported outcomes related to AF ablation were included, such as AF recurrence, AF severity, or quality of life.

Additional file 1: Table S2 provides the detailed search strategy.

### Study selection

We used Endnote X8 database, a reference management software, to organize all studies. All titles and abstracts were reviewed to consider eligible for inclusion. And a full-text evaluation was presented after initial identification.

Eligible studies had to fulfil the following criteria: (1) Clinical trials or observational studies; (2) Study on the relationship between weight loss and outcomes after AF ablation; (3) The patients in this study were adults (age > 18 years), diagnosed with AF, and undergo catheter ablation with weight management;(4) reported the relationship between weight loss and AF recurrence and other outcomes (AF severity, quality, symptoms); (5) The literature reported odds’ ratio (OR), Relative risk (RR), hazard ratio (HR), and the 95% confidence interval (CI) provided available data to calculate the estimation effect for the AF recurrence.

Additionally, we excluded studies with:

1. For multiple reports based on the same data source, we excluded studies with the shorter follow-up time or smaller sample size.
2. Case–control design due to the potential bias.

### Data extraction and quality assessment

Studies were reviewed by two independent authors (X.Z-L and X-L) according to the above inclusion and exclusion criteria. Disagreements were resolved by consensus. Data were extracted by 2 investigators (X.Z-L and X-L), including first author, publication year, country, follow-up time, demographic characteristics (sample size, average age, gender, body mass index (BMI), left atrial diameter, AF type, history of diabetes, history of hypertension, high history of lipemia), study design, data source, methods of weight loss and AF diagnosis, outcomes, corresponding 95% CI and estimate effect, and adjustments.

The quality of the included studies was assessed according to the Newcastle–Ottawa evaluation scale (NOS) and modified Jada scale for cohort and trial, respectively. Scores range from 0 to 9, with NOS scores greater than 7 being considered high quality [16].

### Statistical analysis and bias risk assessment

Review Manager (Version 5.1., The Nordic Cochrane Center, The Cochrane Collaboration, Copenhagen, Denmark, 2011.) and Stata 16.0 (Stata Corp LP, College Station, TX, USA) was used for statistical analysis. The HR was converted to RR, and the supposed OR provided a good estimate of RR. RR were used to combine the estimated effects of random effects models. We estimate the adjusted RR's by calculating the natural logarithm of RR (log [RR]) and its standard error (SE log [RR]). For those studies that did not provide RRs, we calculated crude RR by event and total number. In addition, we performed subgroup analysis stratified by gender, methods of weight loss, and the weight loss time.

For dose–response analysis, we computed summary RRs and 95% CIs for a  10% in weight loss using a random effects model. Study-specific slopes (linear trends) and 95% CIs from the natural logs of the reported RRs and CIs across categories of weight loss by using the method of Greenland and Longnecker [17]. We performed the non-linear dose–response analysis by using the robust error meta-regression method described by Xu et al. [18]. It requires known levels of weight loss and RRs with variance estimates for at least two quantitative exposure categories. If the median or mean weight loss was not provided and reported in ranges, we estimated the midpoint of each category by averaging the lower and upper boundaries of that category. If the highest or lowest category was open-ended, we assumed that the open-ended interval length was the same as the adjacent interval. We used Q statistic and I^2^ statistics to estimate heterogeneity between studies. For Q statistic,a P < 0.10 was regared to indicate significant heterogeneity in the Q statistic. For I^2^ statistics, in I^2^ < 50%, I^2^ at 50% to 75%, I^2^ > 75% were regard as low heterogeneity, moderate heterogeneity and high heterogeneity [16], respectively. Egger’s, Begg’s, or Funnel plot were used to detect publication bias. *P* < 0.05 with two tails is considered statistically significant.

## Results

### Study selection

**Fig. 1:**
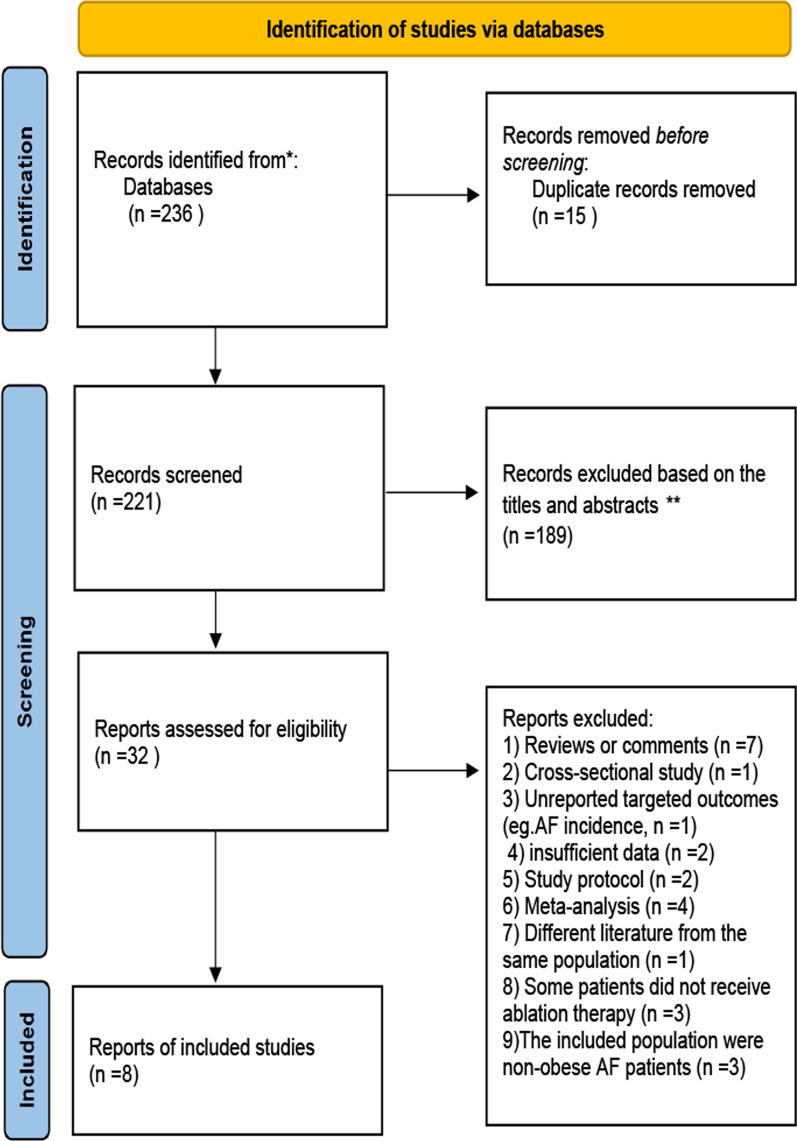
Flowchart of selection of studies that investigated weight loss and outcomes in AF ablation identified in the systematic literature search

As shown in Fig. 1, 236 publications and five conference abstracts were identified in the initial literature search (PubMed = 121; Cochrane Library = 9; Embase = 96; other sources = 10). After excluding duplicates and screening titles and abstracts, 32 articles remained for evaluation by full text. Two studies were based on duplicated populations [19, 20], and studies with a large sample size were included [19]. Eight studies were finally included, and 24 studies were excluded for the following reasons: (1) reviews, comments and case reports (n = 7); (2) cross-sectional studies (n = 1); (3) did not reported targeted outcomes (n = 1); (4) insufficient data (n = 2); (5) study protocols (n = 2); (6) meta-analyses (n = 4); and (7) Literature from the same population (n = 1); (8) patients did not receive ablation therapy (n = 3), (9) The study’s purpose does not meet inclusion criteria (n = 3). Additional file 1: Table S3 describes the excluded studies.

### Study characteristics and quality

Table 1 and Additional file 1: Table S4 summarizes the characteristics of the included studies. One randomized open-labeled,clinical trial [21] with 133 (weight loss = 67, control = 66) patients and seven cohorts with 1150 patients (prospective [22–27] = 6, retrospective [19] = 1) were included. Overall, these studies were published between 2014 and 2021. Sample sizes for the included studies ranged from 90 to 304 with a total of 1150 patients. The average age was 58 to 65 years, and the female proportions was 27.8–44.0%. Follow-up for the entire study ranged from 6 to 60 months. The average BMI of the clinical trial [21] was 34.8kg/m^2^, while the average BMI of the other seven observational studies [19, 22–27] was greater than 30 kg/m^2^. The enrolled patients in the clinical trial [21]are all obese. Meanwhile, the patients in the three observational studies [19, 24, 25] included are all obese; in the observational studies of Bunch et al. [26] and Lau et al. [22], the obesity proportions are 44.4% and 76.0%, respectively, and the obesity proportion in the two observational studies [23, 27] is unclear. Four studies were from the United States [19, 23, 25, 26], two were from the United Kingdom [22, 24], one was from Australia [27], and one was from Germany [21]. Eight studies reported on the recurrence of AF [19, 21–27], three studies reported the burden of AF [21, 25, 27], and one study reported on quality of life [25]. The method of weight loss in the clinical trial [21] was improved lifestyle. The method of weight loss in four observational studies [22, 24, 25, 27]was an improved lifestyle; bariatric surgery was adopted for weight loss in one observational study [19], and the method of weight loss in the other two observational studies [23, 26] was unknown. Weight intervention was used in five of the studies before ablation [19, 22, 24, 25, 27], whereas three studies reported the use of an intervention after ablation [21, 23, 26].  The blank period after ablation of six studies [19, 21, 24–27] is 3 months, and resting two studies [22, 23] were unclear. Four studies defined AF recurrence as an episode for 30 s or more [19, 21, 25, 27], and four studies did not clarify the definition of AF recurrence [22–24, 26]. All AF events were detected by 12-lead ECG, Holter or other forms of dynamic monitoring.

**Table 1:** Main characteristics of the included studies in the meta‐analysis of weight loss and outcomes after AF ablation

Refences (First author, Year, Country/Region)	Source of individuals	Outcomes	Study design	AF diagnosis	Methods of weight loss	N	Age^#^ (year) Male^#^ (%) BMI^#^	Weight loss (%)	RR (95% CI)	Follow-up times (months)	Adjusted covariates
*Clinical trial*											
Gessler 2021, Germany	SORT-AF trial	AF recurrence AF burden	Randomized, open-labeled clinical trial	ECG, ILR interrogation	Lifestyle	67/66	58.7/62.1,64/62, 34.9/34.8	4.5	1.14 (0.37–3.61)	12	NA
*Observational studies*											
Pathak 2014, Australian	ARREST-AF Cohort Study	AF recurrence duration and symptoms of AF	Prospective Cohort	ECG	Lifestyle	149	57.7, 63.8, 32.7	13.1	0.21 (0.09–0.49)	24	Multiple procedures, type of AF, poor BP control
Bunch 2016, USA	LDS Hospital, Intermountain Medical Center	AF recurrence	Prospective Cohort	ECG	NA	288	36.3, 59.3, 36	4.5	0.73 (0.50–1.10)	36	Multivariable adjusted&
Mohanty 2017, USA	St. David’s Medical Center	AF recurrence symptom severity of AF quality of life	Prospective Cohort	ECG	Lifestyle	90	62.7, 72.2, 37.6	20.5	0.89 (0.52–1.53)	12	NA
Donnellan 2019, USA	Cleveland Clinic	AF recurrence	Retrospective Cohort	ECG, Holter	Bariatric surgery	239	64.6, 55.6, 41.1	20.6	0.14 (0.05–0.39)	60	HbA1C, presenting rhythm at the time of ablation, EFV, BMI, LA
Ding 2020, UK	Liverpool Heart and Chest Hospital	AF recurrence	Prospective Cohort	ECG, Holter	Lifestyle	92	64.5, 56.5, 36.0	6	1.85 (0.70–4.90)	12	Sex, type of AF, age
Y Lau 2020, UK	Glasgow Royal Infirmary	AF recurrence	Prospective Cohort	NA	Lifestyle	146	NA, NA, 30.4	4.2	0.33 (0.29–0.85)	6	NA
Shah 2020, USA	The University of Rochester Medical Center	AF recurrence	Prospective Cohort	NA	NA	146	61, 67,31	22	0.29 (0.11–0.80)	12	Multivariable adjusted&

*AF* Atrial fibrillation, *ARREST-AF* Aggressive Risk factor reduction study for atrial fibrillation, *CARDIO-FIT Study* cardio- respiratory fitness, *SORT-AF* Supervised obesity reduction trial for AF, *ILR* Implantation loop recorder, *UK* United Kingdom, *USA* United States of America, *ECG* Electrocardiograph, *BMI* Body mass index, *HbA1C* Hemoglobin A1C, *BS* Bariatric surgery, *EFV* Epicardial fat volume, *LA* Left atrium & unclear the exactly adjusted covariate, *NA* not available

*AF burden was quantified by using the validated Atrial Fibrillation Severity Scale

^#^The first number refers to the loss weight group and the second to the control; one column refers to the mean or median values of the overall cohort

It is generally considered that the quality of the literature with a score of 4–7 on the modified Jada scale is high, while the score of the modified Jada scale of the SORT-AF trial is 4, so the quality of the SORT-AF trial is acceptable (Additional file 1: Table S5). The overall study quality of observational data was acceptable with NOS > 6 for all studies (Additional file 1: Table S6).

### Weight loss and AF recurrence after ablation

**Fig. 2:**
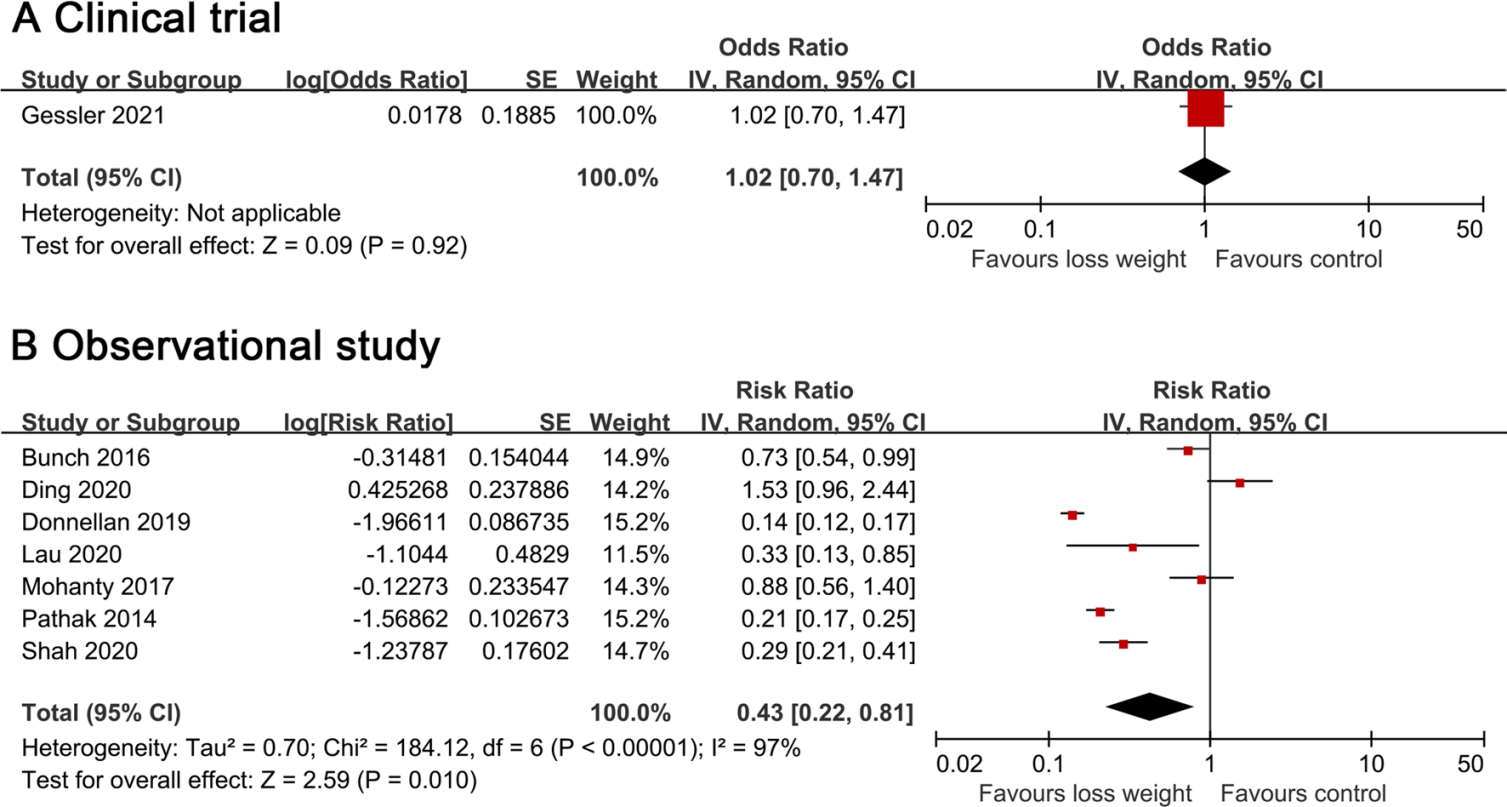
Forest plot of the study of weight loss and AF recurrence after AF ablation in a random-effects model, stratified by study design. Referents for weight loss were the individuals reporting no weight loss or stable weight within the specific study. The diamond indicates the pooled estimate. Red or blue boxes are relative to study size, and the black vertical lines indicate 95% CIs around the effect size estimate

One randomized, open-labeled clinical trial(SORT-AF) [21] and seven cohorts reported weight loss and AF recurrence after ablation [19, 22–27]. In the clinical trial, the intervention group achieved a mean percentage weight loss of 3.91% compared with 0.91% in the control group, demonstrating that weight loss did not significantly reduce the recurrence rate of AF after ablation (OR = 1.02, 95% CI 0.70–1.47) after 12 months of follow-up. Among the observational studies, the mean BMI was over 30 kg/m^2^ in all studies, and six studies were based on patients with obesity [19, 22–25, 27]. Four articles showed a lower AF recurrence rate after ablation of weight loss [19, 22, 23, 27], whereas the remaining three reported no significant association [24–26]. The pooled results of observational studies showed that weight loss in relatively obese patients was associated with a significant risk of AF relapse after ablation (RR = 0.43.95% CI 0.22–0.81) with substantial heterogeneity (Q statistic: *P*  < 0.00001; I^2^ = 97%) [19, 22–27] (Fig. 2).

### Dose–response association of weight loss and AF recurrence

**Fig. 3:**
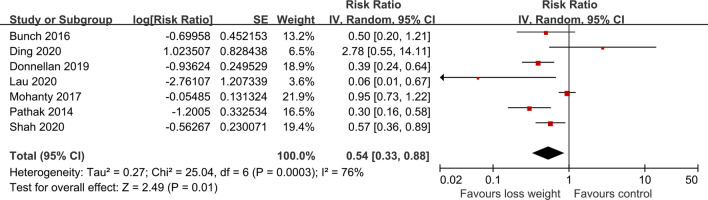
Forest plot of the study-specific RRs for AF recurrence after ablation for every 10% weight loss. Study-specific estimates obtained by the method of Greenland and Longnecker assuming a linear relationship of the RRs to the referent in a random-effects model. Referents for weight loss were the individuals reporting no weight loss or stable weight within the specific study. The diamond indicates the pooled estimate. Red boxes are relative to study size, and the black vertical lines indicate 95% CIs around the effect size estimate

**Fig. 4:**
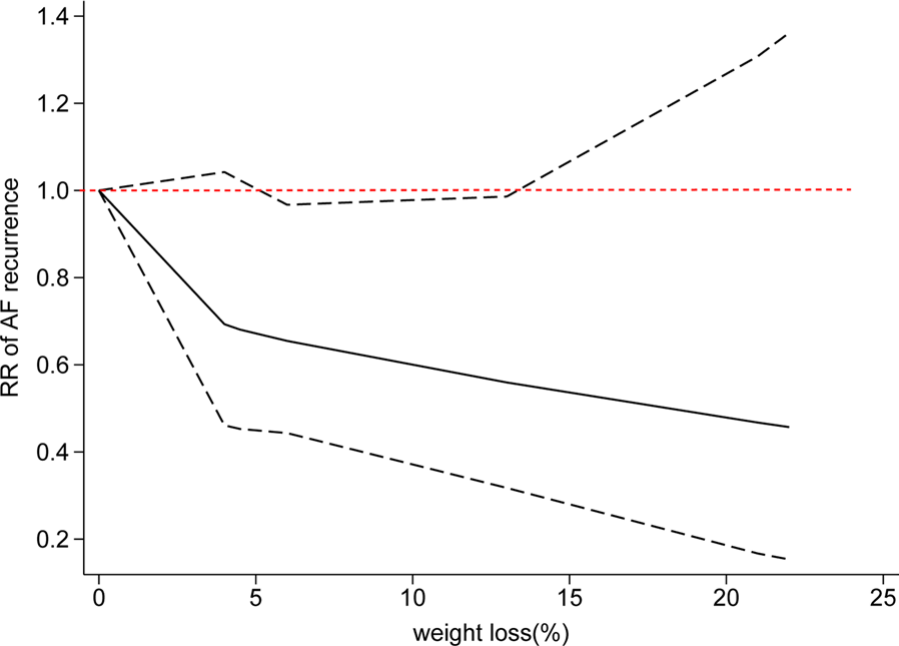
Dose–response association between weight loss and AF recurrence after AF ablation modeled using restricted cubic splines. The bold lines indicate the pooled restricted cubic spline model, and the black dashed line indicates the 95% CIs of the pooled curve

A pooled analysis from seven cohort studies showed a significant association between each 10% weight loss and AF recurrence rates after ablation (RR = 0.54, 95% CI 0.33–0.88) with high statistical heterogeneity (Q statistic: *P* = 0.0003; I^2^ = 76%) [19, 22–27] (Fig. 3). The nonlinear dose–response association was fitted by the restricted cubic splines function, showing a significant trend of inverse association (P_non-linearity_ = 0.27) of AF relapse after ablation with increasing weight loss. However, the results were merely significant at a range of 6–13% weight loss (Fig. 4). Additional file 1: Table S7 summarizes weight loss exposure doses for included studies.

### Subgroup analysis

A pre-defined subgroup analysis stratified by the weight loss method was available. The results showed that bariatric surgery was associated with lower AF relapse after ablation (bariatric surgery, RR = 0.14, 95% CI 0.12–0.17) [19]. However, although a nonsignificant association was noted for lifestyle management (lifestyle management, RR = 0.56, 95% CI 0.18–1.69) [22, 24, 25, 27]. a significant decreasing trend was observed (Additional file 1: Figure S1A). Similar results were found for each 10% reduction in different weight loss method (bariatric surgery [19], RR = 0.39, 95% CI 0.24–0.64, lifestyle management [22, 24, 25, 27], RR = 0.58, 95%: 0.21–1.59), with substantial heterogeneity (Q statistic: *P* < 0.0007; I^2^ = 82%) (Additional file 1: Figure S1B).

Pre-defined subgroup analysis stratified by the weight loss time showed that weight loss was associated with lower AF relapse when patients receive weight management before ablation (RR = 0.41; 95% CI 0.19–0.92). However, when patients received weight management after ablation, the weight loss did not significantly decrease AF recurrence (RR = 0.46, 95% CI 0.19–1.14) (Additional file 1: Figure S2), with no significant subgroup difference (*P* = 0.86) (Additional file 1: Figure S2).

### Sensitivity analysis and publication bias

The sensitivity analysis of leave-one-out methods, omitting crude results, and changing the random model to a fixed model generally produced results consistent with observational studies (Additional file 1: Figure S3). Publication bias and meta-regression were not performed due to the limited number of included studies according to the guidelines (N < 10).

### Weight loss and AF severity, symptoms, and quality of life

The results from SORT-AF showed that no significant difference in the AF burdens were noted among the patients undergoing weight loss intervention and controls (OR = 1.14, 95% CI 0.36–3.61, *P* = 0.81). [21]Evidence from the observational study showed that weight loss was significantly associated with reduced duration and symptoms of AF (*P* < 0.001) [27]. Mohanty et al. [25] showed that AF weight loss significantly improved quality of life (*P* < 0.002) but not the symptom severity of AF (*P* = 0.84).

### GRADE assessments

The GRADE framework indicated moderate certainty in the weight loss summary finding for AF recurrence after ablation based on observational studies (data not shown).

## Discussion

### Major findings

The major findings of the present meta-analysis based on observational studies were as follows: (i) Weight loss is associated with a reduced risk of AF recurrence after ablation. (ii) A  10% reduction in weight loss is associated with a 27% risk of AF recurrence after ablation. (iii) A potential dose–response relationship is noted between weight loss and AF relapse after ablation. To the best of our knowledge, this is the first meta-analysis that reported the dose-response association between weight loss and the risk of AF recurrence after ablation, providing an informative finding for the improvement of outcomes of AF ablation.

Obesity is a well-known independent risk factor for AF. As shown by several studies, obesity is associated with an approximately 50% increase in the prevalence of AF in the general population with a 1-unit increase in BMI increasing the risk of AF by 3% to 4.7% [28, 29]. This association persisted in patients undergoing ablation. Previous studies demonstrated that overweight/obese patients receiving ablation might be associated with worse outcomes, including increased AF recurrence and poor quality of life [30, 31]. Previous observational studies, such as the landmark LEGACY study, showed that long-term sustained weight loss is associated with a significant reduction in the burden of AF and the maintenance of sinus rhythm. A meta-analysis based on post hoc analysis of randomized controlled studies (RCTs) also showed similar results [14]. In the present study, we showed a potential benefit of weight loss on AF recurrence after ablation based on observational studies, adding more recent evidence on this subject. Notably, the SORT-AF trial [20] showed that weight loss based on lifestyle management had a nonsignificant benefit on the AF burden or AF recurrence rate. Several reasons might explain these inconclusive results. First, the SORT-AF weight loss group achieved a mean weight reduction of 4.6 kg (3.91% of their initial body weight), which might not be sufficient to show an effect on outcomes. Regarding the LEGACY study, the results showed the greatest effect on freedom from AF in patients who lost > 10% of their body weight. Second, the number of included patients were also limited, which might make the study underpowered to detect an effect on AF burden. Third, group 1 of the SORT-AF trial had more persistent AF patients, although cox proportional hazards models had adjusted types of AF, but the type of AF in both groups was not comparable. This may be regarded as a significant confounder. Finally, if there had been weight loss during ablation and not afterward, the effect of weight loss might be beneficial. Our dose–response curve also showed a trend but no significant decreased risk of AF recurrence for weight loss < 6%, which reinforced this opinion. Thus, we supposed that weight loss in patients with AF ablation was associated with a lower risk of recurrent AF.

The subgroup analysis of pre-ablative loss weight demonstrated that weight loss was caused by a healthy lifestyle. In this meta-analysis, weight intervention was used in five of the studies before ablation [19, 22, 24, 25, 27], whereas three studies reported the use of an intervention after ablation [21, 23, 26]. The results of the clinical trial reveal that weight loss did not significantly reduce the recurrence rate of AF after ablation (OR = 1.02, 95% CI 0.70–1.47) after 12 months of follow-up [21]. Other two observations studies show that loss weight did not significantly reduce the recurrence rate of AF after ablation (RR = 0.46, 95% CI 0.19–1.14) [23, 26]. There are studies demonstrated that pre-ablative weight loss can enhance insulin sensitivity and improve glycemic control, ameliorating epicardial fat deposition and inflammation [32, 33]. Moreover, pre-ablative weight loss can optimize the outcomes of ablation through the improved obesity-mediated structural remodeling of the atrium [34]. However, our results showed that weight loss was associated with a lower AF relapse trend, but the pre-ablative and post-ablation did not showed a subgroup difference.

The ablation strategy across studies varied. Radiofrequency ablation was used in SORT-AF trial’s [20] and two observation studies [24, 26]. Cryoballoon ablation was used in one observation study [25]. One cohort [23] reported mixed radiofrequency and Cryoballoon ablation. Ablation strategy of the resting three observation studies [21, 22] is unclear. Currently, a Systematic Review and Meta-Analysis [35] showed there was no significant difference of the clinical outcomes between cryoballoon ablation and radiofrequency, and the 2020 ESC guideline [12] of AF indicated that in the first procedure for paroxysmal AF, the outcomes of either strategies are analogous. Therefore, the heterogeneity due to different ablation strategies may not influence our result.

Meanwhile, we must recognize that the prognosis for AF ablation is related to gender. Some studies demonstrate that women might have a higher risk of AF recurrence after ablation than men [36, 37]. Some reasons may account for the sex differences in ablation effectiveness, including lower frequency or delayed referral for ablation, higher atrial fibrosis, older age, a more complex clinical profile, and a higher prevalence of non-pulmonary vein triggers in women [31, 37–39].

### Comparisons with previous studies

Several systematic reviews have shown that weight loss decreases the risk of AF. Jones et al. [40] showed that a 5% loss in body weight was not associated with a significant change in the incidence of AF. Aldaas et al. [41] showed that patients who lost ≥ 10% of their initial body weight had a lower risk of recurrent AF, a reduction in AF burden, and an improvement in AF symptom severity. Similarly, a systematic review demonstrated that weight loss is associated with a lower long-term recurrence of AF after ablative therapy [42]. Our results expand the effect of weight loss on clinical outcomes, including AF severity, quality, symptoms, for AF ablation and further firstly clarify the potential dose–response association.

### Underlying mechanism

There are several potential mechanisms involved in the association between weight loss and AF recurrence after AF ablation. Obesity leads to atrial structure and electrical remodeling, which makes patients prone to AF [43]. This observation is mainly related to the inflammatory response, fibrosis and oxidative stress induced by the increase in atrial fat cells and epicardial fat [1, 44]. Weight loss was shown to reduce the atrial area, improve inflammation, and reduce myocardial fibrosis, which has an important impact on slowing down the structural remodeling of the heart [45]. Furthermore, obesity is an independent risk factor for hypertension, diabetes, and obstructive sleep apnea syndrome. These comorbidities are common in patients with obesity, and weight loss can reduce blood pressure, improve insulin sensitivity, and blood sugar control, improve sleep apnea syndrome, and reduce all these comorbidities.

### Policy implications and further research

The 2020 ESC Guidelines set the weight loss target at ≥ 10% weight reduction for patients with BMI > 27 kg/m^2^ for the improvement of AF ablation [12]. However, whether a smaller magnitude of weight loss benefits prognosis after ablation remains unknown. Our study showed that a 10% reduction in weight is associated with a decreased risk of AF recurrence based on observational studies. Furthermore, a potential linear inverse dose–response association is noted between weight loss and reduced AF recurrence. Yet, we acknowledged that the dose–response association should be considered exploratory due to the limited study numbers and intrinsic limitations of the observational design. Further trials are needed to confirm the effect of a smaller magnitude of weight loss.

Current guidelines do not have specific comments on bariatric surgery on AF ablation. Our results showed that bariatric surgery has a significant effect on preventing AF relapse after ablation, but only one study was included. Nevertheless, considering the results of the LEGACY study, we hypothesized that bariatric surgery might be more appropriate and beneficial in those with mortality obesity [46], especially those with decreased physical ability. Nevertheless, the decision for bariatric surgery before ablation should be evaluated in combination with a clinical basis; the potential benefit and risk of weight loss need to be balanced. Increasing the duration of AF has adverse effects on ablation success rates. This procedure would necessitate an at least a 6-months delay in ablation, leading to the progression of AF with known worsening of success rates. In the subgroup of lifestyle management, the results were nonsignificant, but the statistical power might be limited by the small sample size.

According to the GRADE framework, the weight loss summary finding for AF recurrence after ablation based on observational studies has moderate certainty. Thus, we recommend AF patients who are obese may loss weight to maximize the effect of AF ablation.

Although we showed a significant benefit of weight loss on AF recurrence, specifics regarding the severity of AF and the burden of AF of weight loss are not clear. Evidence from the SORT-AF trial did not achieve a significant benefit for the AF burden. The evidence based on observational studies is also inclusive. However, emerging evidence has shown that benefits of weight loss on blood pressure, glucose, insulin resistance, low-density lipoprotein, cardiovascular events, and mortality. We hypothesize that this benefit might also persist in AF ablation, which may have been even clearer in further trials with effective designs, such as a larger group of patients.

Finally, several issues remain unresolved. For example, men might have a better outcome than women regarding AF ablation [47]. Furthermore, post hoc analysis from SORT-AF showed that weight loss was associated with a more pronounced reduction in the AF recurrence rate in persistent AF patients than in paroxysmal AF patients [47]. These observations highlight the complexity of the physiology of AF, which calls for more clinical studies.

### Limitations

Several limitations are noted for any observational study. First, our main findings were based on observational studies, the numbers of included studies were limited, and measurement and unmeasured bias cannot be completely excluded. However, the cohort studies reduced the selection bias. Second, we did not assess the association between weight loss and hard outcomes, such as stroke, cardiovascular death, or all-cause death. As we previously described, overweight or class I obesity patients seem to have better survival outcomes than normal-weight individuals in patients with AF, a phenomenon that is known as the “obesity paradox” [16, 48]. The LEGACY results showed better survival outcomes in patients with obesity undergoing bariatric surgery.

However, direct evidence for the association between weight loss and AF ablation involving hard outcomes is limited. Third, significant heterogeneity was noted in the main results, which might be derived from the difference in baseline characteristics, antiarrhythmic medications, time of weight loss, and different types of weight loss interventions (lifestyle management or bariatric surgery).

## Conclusion

Our meta-analysis suggests that weight loss is associated with a decreased recurrence rate of AF after ablation, with moderate certainty. The effect of weight loss on AF burden, and quality of life in patients after AF ablation needs to be further studied.

## Supplementary Information

**Additional file 1**.** Table S1**. Article content checklist.** Table S2**. Detailed description of the search strategy.** Table S3**. Studies excluded (n=24) with reasons.** Table S4**. Main Clinical characteristics of the included studies in the meta‐analysis.** Table S5**. Quality assessment of included randomized, open-labeled clinical trial.** Table S6**. Quality assessment of included studies.** Table S7**. Summary of weight loss exposure dose for the included studies.** Figure S1**. Subgroup analysis of loss weight on AF recurrence after ablation, stratified by method of weight loss.** Figure S2**. Subgroup analysis of loss weight on AF recurrence after ablation, stratified by pre-ablative.** Figure S3**. Sensitivity analysis of loss weight on AF recurrence after ablation.

## Data Availability

The raw data required to reproduce these findings are available from the corresponding author.

## Abbreviations

AF: Atrial fibrillation
RRs: Relative risks
CI: Confidence interval
OR: Odds’ ratio
HR: Hazard ratio
NOS: Newcastle–Ottawa evaluation scale
RCTs: Randomized controlled study
BMI: Body mass index
