# Supplementary material for: Association between weight loss and outcomes in patients undergoing atrial fibrillation ablation: a systematic review and dose–response meta-analysis
Source: Nutr Metab (Lond). 2023 Jan 31;20:5. doi: 10.1186/s12986-023-00724-5 (PMC9890842; doi:10.1186/s12986-023-00724-5)
Supplement: Supplementary file 1 — Additional file 1. Table S1. Article content checklist. Table S2. Detailed description of the search strategy. Table S3. Studies excluded (n=24) with reasons. Table S4. Main Clinical characteristics of the included studies in the meta‐analysis. Table S5. Quality assessment of included randomized, open-labeled clinical trial. Table S6. Quality assessment of included studies. Table S7. Summary of weight loss exposure dose for the included studies. Figure S1. Subgroup analysis of loss weight on AF recurrence after ablation, stratified by method of weight loss. Figure S2. Subgroup analysis of loss weight on AF recurrence after ablation, stratified by pre-ablative. Figure S3. Sensitivity analysis of loss weight on AF recurrence after ablation. [file 12986_2023_724_MOESM1_ESM.docx]

**SUPPLEMENTAL MATERIALS**

**Association of weight loss and outcomes in atrial fibrillation patients post ablation, a systematic review and meta-analysis**

**Table S1: Article content checklist**

**TableS2**: **Detailed description of the search strategy**

**Table S3: Studies excluded (n=24) with reasons**

**Table S4. Main Clinical characteristics of the included studies in the meta‐analysis**

**Table S5**. **Quality assessment of included randomized, open-labeled clinical trial**

**Table S6** **Quality assessment of included studies**

**Table S7. Summary of weight loss exposure dose for the included studies**

**Figure S1.** [**Subgroup**](javascript:;) [**analysis**](javascript:;) **of loss weight on AF recurrence after ablation, stratified by method of weight loss**

**Figure S2.** [**Subgroup**](javascript:;) [**analysis**](javascript:;) **of loss weight on AF recurrence after ablation, stratified by pre-ablative**

**Figure S3.** [**Sensitivity**](javascript:;) [**analysis**](javascript:;) **of loss weight on AF recurrence after ablation**

**Table S1: Article content checklist**

| **Section and Topic** | **Item #** | **Checklist item** | **Location where item is reported** |
| --- | --- | --- | --- |
| **TITLE** | | |  |
| Title | 1 | Identify the report as a systematic review. | 1 |
| **ABSTRACT** | | |  |
| Abstract | 2 | See the PRISMA 2020 for Abstracts checklist. | 1 |
| **INTRODUCTION** | | |  |
| Rationale | 3 | Describe the rationale for the review in the context of existing knowledge. | 2 |
| Objectives | 4 | Provide an explicit statement of the objective(s) or question(s) the review addresses. | 2-3 |
| **METHODS** | | |  |
| Eligibility criteria | 5 | Specify the inclusion and exclusion criteria for the review and how studies were grouped for the syntheses. | 4 |
| Information sources | 6 | Specify all databases, registers, websites, organisations, reference lists and other sources searched or consulted to identify studies. Specify the date when each source was last searched or consulted. | 3 |
| Search strategy | 7 | Present the full search strategies for all databases, registers and websites, including any filters and limits used. | 3-4 |
| Selection process | 8 | Specify the methods used to decide whether a study met the inclusion criteria of the review, including how many reviewers screened each record and each report retrieved, whether they worked independently, and if applicable, details of automation tools used in the process. | 4 |
| Data collection process | 9 | Specify the methods used to collect data from reports, including how many reviewers collected data from each report, whether they worked independently, any processes for obtaining or confirming data from study investigators, and if applicable, details of automation tools used in the process. | 4-5 |
| Data items | 10a | List and define all outcomes for which data were sought. Specify whether all results that were compatible with each outcome domain in each study were sought (e.g. for all measures, time points, analyses), and if not, the methods used to decide which results to collect. | 4 |
|  | 10b | List and define all other variables for which data were sought (e.g. participant and intervention characteristics, funding sources). Describe any assumptions made about any missing or unclear information. | 4 |
| Study risk of bias assessment | 11 | Specify the methods used to assess risk of bias in the included studies, including details of the tool(s) used, how many reviewers assessed each study and whether they worked independently, and if applicable, details of automation tools used in the process. | 5 |
| Effect measures | 12 | Specify for each outcome the effect measure(s) (e.g. risk ratio, mean difference) used in the synthesis or presentation of results. | 4 |
| Synthesis methods | 13a | Describe the processes used to decide which studies were eligible for each synthesis (e.g. tabulating the study intervention characteristics and comparing against the planned groups for each synthesis (item #5)). | 5 |
|  | 13b | Describe any methods required to prepare the data for presentation or synthesis, such as handling of missing summary statistics, or data conversions. | 5 |
|  | 13c | Describe any methods used to tabulate or visually display the results of individual studies and syntheses. | 5 |
|  | 13d | Describe any methods used to synthesize results and provide a rationale for the choice(s). If meta-analysis was performed, describe the model(s), method(s) to identify the presence and extent of statistical heterogeneity, and software package(s) used. | 5 |
|  | 13e | Describe any methods used to explore possible causes of heterogeneity among study results (e.g. subgroup analysis, meta-regression). | 5 |
|  | 13f | Describe any sensitivity analyses conducted to assess the robustness of the synthesized results. | 5 |
| Reporting bias assessment | 14 | Describe any methods used to assess risk of bias due to missing results in a synthesis (arising from reporting biases). | 6 |
| Certainty assessment | 15 | Describe any methods used to assess certainty (or confidence) in the body of evidence for an outcome. | 6 |
| **RESULTS** | | |  |
| Study selection | 16a | Describe the results of the search and selection process, from the number of records identified in the search to the number of studies included in the review, ideally using a flow diagram. | 6-7 |
|  | 16b | Cite studies that might appear to meet the inclusion criteria, but which were excluded, and explain why they were excluded. | 7 |
| Study characteristics | 17 | Cite each included study and present its characteristics. | 7-8 |
| Risk of bias in studies | 18 | Present assessments of risk of bias for each included study. | 8 |
| Results of individual studies | 19 | For all outcomes, present, for each study: (a) summary statistics for each group (where appropriate) and (b) an effect estimate and its precision (e.g. confidence/credible interval), ideally using structured tables or plots. | 9 |
| Results of syntheses | 20a | For each synthesis, briefly summarise the characteristics and risk of bias among contributing studies. | 9 |
|  | 20b | Present results of all statistical syntheses conducted. If meta-analysis was done, present for each the summary estimate and its precision (e.g. confidence/credible interval) and measures of statistical heterogeneity. If comparing groups, describe the direction of the effect. | 9 |
|  | 20c | Present results of all investigations of possible causes of heterogeneity among study results. | 10 |
|  | 20d | Present results of all sensitivity analyses conducted to assess the robustness of the synthesized results. | 10 |
| Reporting biases | 21 | Present assessments of risk of bias due to missing results (arising from reporting biases) for each synthesis assessed. | 10 |
| Certainty of evidence | 22 | Present assessments of certainty (or confidence) in the body of evidence for each outcome assessed. | 11 |
| **DISCUSSION** | | |  |
| Discussion | 23a | Provide a general interpretation of the results in the context of other evidence. | 10-12 |
|  | 23b | Discuss any limitations of the evidence included in the review. | 12-13 |
|  | 23c | Discuss any limitations of the review processes used. | 15-16 |
|  | 23d | Discuss implications of the results for practice, policy, and future research. | 13-15 |
| **OTHER INFORMATION** | | |  |
| Registration and protocol | 24a | Provide registration information for the review, including register name and registration number, or state that the review was not registered. | 3 |
|  | 24b | Indicate where the review protocol can be accessed, or state that a protocol was not prepared. | 3 |
|  | 24c | Describe and explain any amendments to the information provided at registration or in the protocol. | 3 |
| Support | 25 | Describe sources of financial or non-financial support for the review, and the role of the funders or sponsors in the review. | 1 |
| Competing interests | 26 | Declare any competing interests of review authors. | 1 |
| Availability of data, code and other materials | 27 | Report which of the following are publicly available and where they can be found template data collection forms; data extracted from included studies; data used for all analyses; analytic code; any other materials used in the review. | 1 |

**TableS2**: **Detailed description of the search strategy**

**PubMed**

| Search | Query |
| --- | --- |
| #1 | atrial Fibrillation |
| #2 | atrial flutter |
| #3 | atrial tachycardia |
| #4 | ablation |
| #5 | weight loss |
| #6 | bariatric surgery |
| #7 | #1OR #2 OR #3 |
| #8 | #5 OR #7 |
| #9 | #4 AND #7 AND#8 |

**Embase**

| Search | Query |
| --- | --- |
| #1 | 'atrial fibrillation'/exp |
| #2 | ' atrial flutter '/exp |
| #3 | ' atrial tachycardia '/exp |
| #4 | 'ablation therapy'/exp |
| #5 | 'loss weight' |
| #6 | ' bariatric surgery '/exp |
| #7 | #1OR #2 OR #3 |
| #8 | #5 OR #7 |
| #9 | #4 AND #7 AND#8 |

**Cochrane**

| Search | Query |
| --- | --- |
| #1 | atrial Fibrillation |
| #2 | atrial flutter |
| #3 | atrial tachycardia |
| #4 | ablation |
| #5 | weight loss |
| #6 | bariatric surgery |
| #7 | #1OR #2 OR #3 |
| #8 | #5 OR #7 |
| #9 | #4 AND #7 AND#8 |

**Table S3: Studies excluded (n=24) with reasons**

| **Studies excluded** | **Reasons** |
| --- | --- |
| Alings 2013 ^(1)^ | This is a study protocol |
| Pathak 2015 ^(2)^ | AF patients have not been treated with ablation surgery |
| Pathak 2015(3) | Some patients did not receive ablation therapy |
| Boriani 2015 ^(4)^ | The study's purpose does not meet inclusion criteria. |
| Fioravanti 2017 ^(5)^ | AF patients have not been treated with ablation surgery |
| Lau 2017 ^(6)^ | This is a review |
| Kirchhof 2017 ^(7)^ | This is a review |
| Goudis 2017 ^(8)^ | This is a review |
| Zakeri 2017 ^(9)^ | This is a review |
| Münkler 2018 ^(10)^ | This is a case report |
| Ricci 2018 ^(11)^ | This is a meta-analysis |
| Sunkara 2018 (12) | Study results do not include AF recurrence |
| Donnellan 2019^(13)^ | Different literature from the same population |
| Packer 2019 ^(14)^ | This is a review |
| Tregoning 2019 ^(15)^ | Insufficient data |
| Cherian 2020 ^(16)^ | This is a review |
| Estes 2020 ^(17)^ | This is a comment |
| Kiuchi 2020 ^(18)^ | This is a comment |
| Akhtar 2021 ^(19)^ | This is a meta-analysis |
| D'Souza 2021^(20)^ | Cross-sectional study |
| Oesterle 2021(21) | This is a meta-analysis |
| Peigh 2021(22) | Insufficient data |
| Kewcharoen 2021(23) | This is a meta-analysis |
| Štolbová 2021(24) | This is a study protocol |

1. Alings M, Smit MD, Moes ML, Crijns H, Tijssen JGP, Brugemann J, et al. Routine versus aggressive upstream rhythm control for prevention of early atrial fibrillation in heart failure: background, aims and design of the RACE 3 study. Netherlands heart journal. 2013;21(7‐8):354‐63.

2. Pathak RK, Middeldorp ME, Meredith M, Mehta AB, Mahajan R, Wong CX, et al. Long-Term Effect of Goal-Directed Weight Management in an Atrial Fibrillation Cohort: A Long-Term Follow-Up Study (LEGACY). Journal of the American College of Cardiology. 2015;65(20):2159-69.

3. Pathak RK, Elliott A, Middeldorp ME, Meredith M, Mehta AB, Mahajan R, et al. Impact of CARDIOrespiratory FITness on Arrhythmia Recurrence in Obese Individuals With Atrial Fibrillation: The CARDIO-FIT Study. Journal of the American College of Cardiology. 2015;66(9):985-96.

4. Boriani G, Savelieva I, Dan GA, Deharo JC, Ferro C, Israel CW, et al. Chronic kidney disease in patients with cardiac rhythm disturbances or implantable electrical devices: clinical significance and implications for decision making-a position paper of the European Heart Rhythm Association endorsed by the Heart Rhythm Society and the Asia Pacific Heart Rhythm Society. Europace. 2015;17(8):1169-96.

5. Fioravanti F, Brisinda D, Sorbo AR, Lombardi G, La Brocca L, Fenici R. Compliance in weight control reduces atrial fibrillation worsening: A retrospective cohort study. Nutrition, Metabolism and Cardiovascular Diseases. 2017;27(8):711-6.

6. Lau DH, Nattel S, Kalman JM, Sanders P. Modifiable Risk Factors and Atrial Fibrillation. Circulation. 2017;136(6):583-96.

7. Kirchhof P, Calkins H. Catheter ablation in patients with persistent atrial fibrillation. Eur Heart J. 2017;38(1):20-6.

8. Goudis CA, Vasileiadis IE, Liu T. Epicardial adipose tissue and atrial fibrillation: pathophysiological mechanisms, clinical implications, and potential therapies. Current Medical Research and Opinion. 2018;34(11):1933-43.

9. Zakeri R, Van Wagoner DR, Calkins H, Wong T, Ross HM, Heist EK, et al. The burden of proof: The current state of atrial fibrillation prevention and treatment trials. Heart rhythm. 2017;14(5):763-82.

10. Münkler P, Wutzler A, Attanasio P, Huemer M, Parwani AS, Haverkamp W, et al. Ventricular tachycardia (VT) storm after cryoballoon-based pulmonary vein isolation. American Journal of Case Reports. 2018;19:1078-82.

11. Ricci C, Gervasi F, Gaeta M, Smuts CM, Schutte AE, Leitzmann MF. Physical activity volume in relation to risk of atrial fibrillation. A non-linear meta-regression analysis. Eur J Prev Cardiol. 2018;25(8):857-66.

12. Sunkara B, Li Y, Siontis K, Yokokawa M, Latchamsetty R, Ghanbari H, et al. Effect of maintenance of sinus rhythm after catheter ablation on body weight in patients with atrial fibrillation. Journal of the American College of Cardiology. 2018;71(11).

13. Donnellan E, Wazni O, Kanj M, Hussein A, Baranowski B, Lindsay B, et al. Outcomes of Atrial Fibrillation Ablation in Morbidly Obese Patients Following Bariatric Surgery Compared With a Nonobese Cohort. Circulation Arrhythmia and electrophysiology. 2019;12(10):e007598.

14. Packer M. Disease-treatment interactions in the management of patients with obesity and diabetes who have atrial fibrillation: The potential mediating influence of epicardial adipose tissue. Cardiovascular Diabetology. 2019;18(1).

15. Tregoning DM, Methachittiphan N, Kroman AM, Kulandhaisamy S, Sarairah S, Linge A, et al. WEIGHT FLUCTUATION IS ASSOCIATED WITH RECURRENCE OF ATRIAL FIBRILLATION FOLLOWING CATHETER ABLATION. Heart rhythm. 2019;16(5):522.

16. Cherian TS, Callans DJ. Recurrent Atrial Fibrillation After Radiofrequency Ablation: What to Expect. Cardiac electrophysiology clinics. 2020;12(2):187-97.

17. Estes NAM, Jain SK. Risk Factor Modification for Atrial Fibrillation: An Ounce of Prevention. JACC: Clinical Electrophysiology. 2020;6(10):1288-90.

18. Kiuchi MG, Chen S, Carnagarin R, Matthews VB, Schlaich MP. Does sympathetic hyperactivity adversely impact on the effect of pre-ablation bariatric surgery and atrial fibrillation recurrence in morbidly obese patients undergoing atrial fibrillation ablation? Europace. 2020;22(3):506.

19. Akhtar KH, Beard C, Jafry A, Clifton S, Reese J, Asad ZUA. EFFECT OF MODEST (> 10%) WEIGHT LOSS ON RECURRENCE OF ATRIAL FIBRILLATION AFTER CATHETER ABLATION: A SYSTEMATIC REVIEW AND META-ANALYSIS. Journal of the American College of Cardiology. 2021;77(18):346.

20. D'Souza S, Elshazly MB, Dargham SR, Donnellan E, Asaad N, Hayat S, et al. Atrial fibrillation catheter ablation complications in obese and diabetic patients: Insights from the US Nationwide Inpatient Sample 2005–2013. Clinical Cardiology. 2021;44(8):1151-60.

21. Oesterle A, Giancaterino S, Van Noord MG, Pellegrini CN, Fan D, Srivatsa UN, et al. B-PO02-141 EXERCISE TRAINING REDUCES ATRIAL FIBRILLATION RECURRENCE AND BURDEN: A META-ANALYSIS OF RANDOMIZED CONTROLLED TRIALS. Heart rhythm. 2021;18(8):S155.

22. Peigh G, Wasserlauf J, Vogel K, Kaplan RM, Pfenniger A, Marks D, et al. Impact of pre-ablation weight loss on the success of catheter ablation for atrial fibrillation. J Cardiovasc Electrophysiol. 2021;32(8):2097-104.

23. Kewcharoen J, Techorueangwiwat C, Kanitsoraphan C, Leesutipornchai T, Akoum N, Bunch TJ, et al. High-power short duration and low-power long duration in atrial fibrillation ablation: A meta-analysis. J Cardiovasc Electrophysiol. 2021;32(1):71-82.

24. Štolbová K, Novodvorský P, Jakubíková I, Dvořáková I, Mráz M, Wichterle D, et al. Effect of Complex Weight-Reducing Interventions on Rhythm Control in Obese Individuals with Atrial Fibrillation Following Catheter Ablation: A Study Protocol. Advances in Therapy. 2021;38(4):2007-16.

**Table S4. Main Clinical characteristics of the included studies in the meta‐analysis**

| **Refences**  **(First author, Year, Country/Region)** | **ablation therapy** | **Female**  **(%)** | **Obesity (%)**  **(BMI≥30 kg/m^2^)** | **Non paroxysmal (%)** | **LA diameter(cm)** | **Hypertension (%)** | **Coronary artery**  **disease (%)** |
| --- | --- | --- | --- | --- | --- | --- | --- |
| **Clinical trials** |  |  |  |  |  |  |  |
| Gessler 2021, Germany | radiofrequency ablation | 36/38 | 100/100 | 45/32 | 75/67 | 54/58 | 10/14 |
| **Cohorts** |  |  |  |  |  |  |  |
| Pathak 2014, Australian | radiofrequency ablation | 36.2 | NA | 40.3 | 43.9 | 84.6 | 13.4 |
| Bunch 2016，USA | Cryoballoon ablation | 39.5 | 44.4 | 51.7 | NA | NA | NA |
| Mohanty 2017, America | radiofrequency ablation | 27.8 | 100 | 100 | 47 | 60 | 22.2 |
| Donnellan 2019, America | NA | 44.4 | 100 | 61.5 | 44.8 | 79.5 | 13.8 |
| Ding 2020, United Kingdom | radiofrequency ablation, Cryoballoon ablation | 43.5 | 100 | 56.5 | 41.8 | NA | NA |
| Y Lau 2020, United Kingdom | NA | NA | 76 | 25.3 | NA | NA | NA |
| Shah 2020, USA* | NA | 33.0 | NA | 32 | NA | NA | NA |

The first number refers to the loss weight group and the second to the control, LA: Left atrium, AF: atrial fibrillation.

**Table S5**. Quality assessment of included randomized, open-labeled clinical trial

| Author  (Publication Year, country) |  | |  | | |  | | Modified JADA Scale | | | | | | | | |
| --- | --- | --- | --- | --- | --- | --- | --- | --- | --- | --- | --- | --- | --- | --- | --- | --- |
|  | random sequences generation | | | | Allocation concealment | | | | | | Blinding | | | Incomplete outcome  data | | Total |
|  | a | b | | c | d | | e | | f | g | h | i | j | k | l |  |
| Gessler 2021, Germany* | 0 | 1 | | 0 | 0 | | 0 | | 0 | 0 | 2 | 0 | 0 | 1 | 0 | 4 |

a appropriate: data generated by computer or similar methods (2 grade).

b unknown: randomized trials but did not describe the method of randomization (1 grade).

c inappropriate: alternate allocation method (0grade).

d appropriate: neither clinicians nor patients are aware of the assignment sequence (2 grade).

e unknown: the trial only shows the use of random assignment, but the exact method is unclear (1 grade).

f inappropriate: the method used is predictable (0grade).

g unused (0grade).

h appropriate: take the exact same comfort method (2 grade).

i unknown: the exact method is unclear (1 grade)

j inappropriate: not double-blind (0grade)

k described the reasons for withdrawal (1 grade)

l no reason for withdrawal described (0grade)

**Table S6** **Quality assessment of included studies**

| Author  (Publication Year) | Newcastle-Ottawa Scale | | | | | | | | | |
| --- | --- | --- | --- | --- | --- | --- | --- | --- | --- | --- |
|  | Selection | | | Comparability | | | Outcome | | | Total |
|  | a | b | c | d | e | f | g | h | i |  |
| Pathak 2014, Australia | 1 | 1 | 1 | 1 | 1 | 1 | 1 | 0 | 1 | 8 |
| Bunch 2016, USA | 1 | 1 | 1 | 1 | 0 | 0 | 1 | 1 | 1 | 7 |
| Mohanty2017, USA | 1 | 1 | 1 | 1 | 1 | 1 | 1 | 1 | 1 | 9 |
| Donnellan 2019, USA | 1 | 1 | 1 | 1 | 1 | 0 | 1 | 1 | 1 | 8 |
| Y Lau 2020, UK | 1 | 1 | 1 | 1 | 1 | 1 | 1 | 1 | 0 | 8 |
| Shah 2020, USA | 1 | 1 | 1 | 1 | 1 | 0 | 1 | 1 | 1 | 8 |
| Ding 2020, UK | 1 | 1 | 1 | 1 | 1 | 1 | 1 | 1 | 1 | 9 |

1. Representativeness of the exposed cohort.
2. Selection of the non-exposed cohort.
3. Ascertainment of exposure.
4. Demonstration that outcome of interest was not present at start of study.
5. Comparability of cohorts on the basis of the design or analysis (adjusted for age).
6. Comparability of cohorts on the basis of the design or analysis (adjusted for any other factor).
7. Assessment of outcome.
8. Was follow-up long enough for outcomes to occur. (1 years for AF recurrence).
9. Adequacy of follow-up of cohorts.

**Table S7. Summary of weight loss exposure dose for the included studies**

| Author, year, country | Weight loss exposure (dose^*^ in %) | RRs (95%CI) |
| --- | --- | --- |
| Clinical trials |  |  |
| Gessler 2021, Germany | weight loss of < 4% | Reference |
|  | weight loss of > 4% | 1.143 (0.369–3.613) |
| Cohorts |  |  |
| Pathak 2014, Australian | weight loss of < 1% | Reference |
|  | weight loss of > 10% | 0.21(0.09- 0.49) |
| Bunch 2016，USA | weight loss of > 3% | Reference |
|  | maintained weight ± 3 % | 1.37 (0.91, 2.02) |
|  | weight gained > 3% | 1.27 (0.85, 1.85) |
| Mohanty 2017, America | maintained weight | Reference |
|  | weight loss of > 10% | 0.89(0.52-1.53) |
| Donnellan 2019, USA | weight loss of < 5% | Reference |
|  | weight loss of >10% | 0.14 (0.05–0.39) |
| Y Lau 2020, UK | weight gain of > 3% | Reference |
|  | weight loss of > 5% | 0.33(0.13-0.85) |
| Shah 2020, USA | weight gain -0.74% to 16.42% | Reference |
|  | weight loss of 0.77 % to 22% | 0.29(0.11‐0.80) |
| Ding 2020, UK | weight loss of > 10% | Reference |
|  | weight loss of < 10% | 0.32(0.12-0.89) |

**
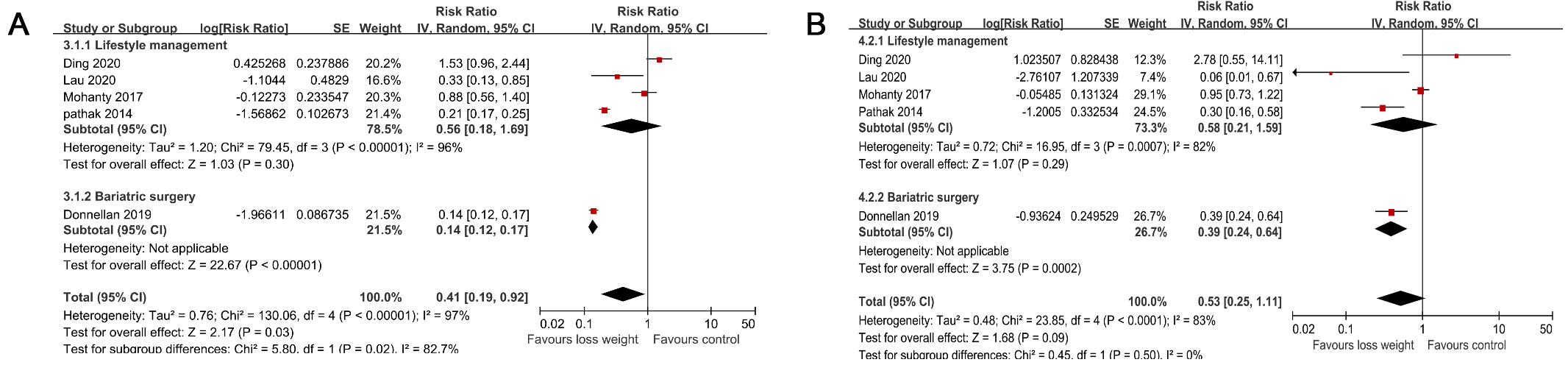
**

**Figure S1.** [**Subgroup**](javascript:;) [**analysis**](javascript:;) **of loss weight on AF recurrence after ablation, stratified by method of weight loss**

# A: Category analysis; right panel: B: per 5% weight loss

The diamond indicates the pooled estimate. Gray boxes are relative to study size, and the black vertical lines indicate 95% CIs around the effect size estimate.


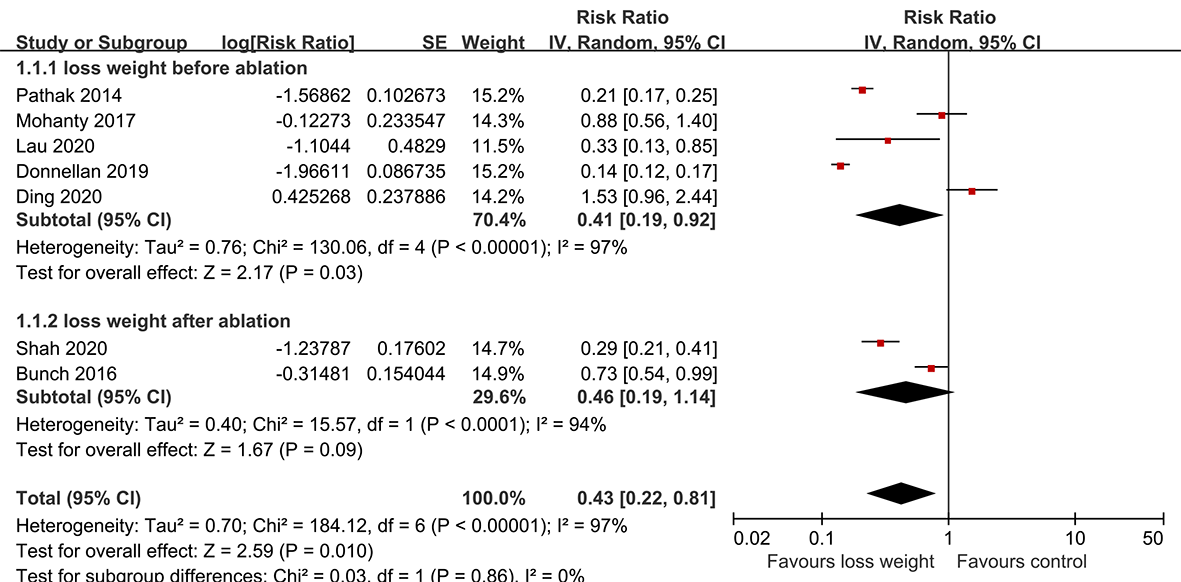


**Figure S2.** [**Subgroup**](javascript:;) [**analysis**](javascript:;) **of loss weight on AF recurrence after ablation, stratified by pre-ablative**

The diamond indicates the pooled estimate. Gray boxes are relative to study size, and the black vertical lines indicate 95% CIs around the effect size estimate.

#
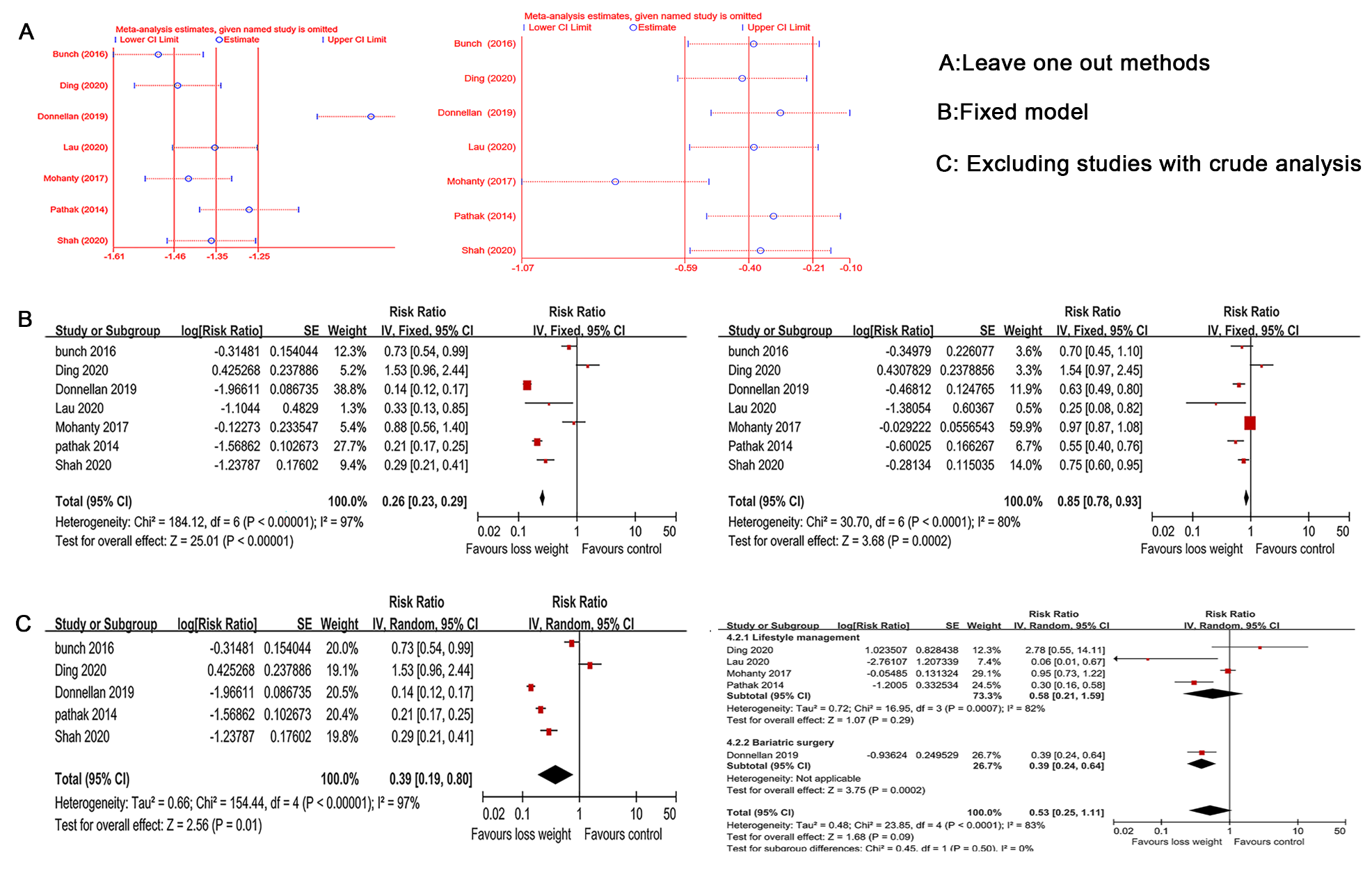


**Figure S3.** [**Sensitivity**](javascript:;) [**analysis**](javascript:;) **of loss weight on AF recurrence after ablation**

# A: Leave one out methods, left panel: category analysis; right panel: per 5% weight loss

# B: By changing random model to Fixed model, left panel: category analysis; right panel: per 5% weight loss

# C: Excluding studies with crude analysis, left panel: category analysis; right panel: per 5% weight loss
